# From synapse to sheath: how OPCs couple neural activity to adaptive myelination and behavioral plasticity

**DOI:** 10.3389/fcell.2026.1966652

**Published:** 2026-09-28

**Authors:** Rui Tan, Wenwen Lu, Aifen Yang, Junqing Du, Xiaofeng Xu, Xiaofeng Zhao, Mengsheng Qiu, Yu Tian

**Affiliations:** Zhejiang Key Laboratory of Organ Development and Regeneration, College of Life and Environmental Sciences, Hangzhou Normal University, Hangzhou, China

**Keywords:** activity-dependent myelination, behavioral plasticity, demyelinating disease, myelin plasticity, oligodendrocyte precursor cells

## Abstract

Oligodendrocyte precursor cells (OPCs) are abundant progenitor cells in the central nervous system. Unlike mature oligodendrocytes, OPCs form functional synapses with neurons and respond to both glutamatergic and GABAergic signals. Glutamate activates calcium-permeable AMPA receptors on OPCs, generating local Ca^2+^ signals that regulate myelin sheath formation and stabilization. In parallel, GABAergic signaling modulates OPC proliferation and differentiation through both calcium-dependent and transcriptional mechanisms. Activity-dependent myelination driven by OPCs contributes directly to neural circuit plasticity and behavioral adaptation, including motor learning, spatial memory, fear extinction, and social experience-dependent brain development. Disruption of the OPC–synapse–myelin axis impairs remyelination and circuit function, contributing to neurological and psychiatric disorders such as multiple sclerosis, schizophrenia, and PTSD. In this review, we critically examine neuron–OPC communication across four interconnected levels: synaptic signaling, cellular fate regulation, myelin plasticity, and behavior. We highlight key limitations in the current evidence, including controversies over the functional role of OPC synapses in the adult brain, unclear mechanisms underlying myelin plasticity, and the largely correlative nature of human neuroimaging data. We argue that the field must move beyond descriptive observations toward rigorous mechanistic studies of causality, with particular attention to region- and cell-type-specific heterogeneity.

## 1 Introduction

A central tenet of modern neuroscience holds that adaptive behavior emerges from the use-dependent modification of neural circuits. Classically, this plasticity was attributed predominantly to changes at chemical synapses between neurons, including long-term potentiation, depression, and structural remodeling of dendritic spines. However, converging evidence over the past 2 decades has revealed a parallel glial arm of this adaptive machinery, namely, the activity-dependent regulation of myelin mediated through synaptic communication between neurons and oligodendrocyte precursor cells (OPCs).

The discovery that OPCs, the NG2+/PDGFRα+ progenitors distributed throughout grey and white matter, form functional synaptic contacts with neurons (Bergles et al., 2000) raised a deceptively simple question: why do progenitor cells need synapses? The answer, now supported by an extensive body of evidence, is that OPCs use synaptic and extrasynaptic neurotransmitter signals alongside activity-dependent paracrine factors, as real-time readouts of local circuit activity and transduce this information into decisions about whether to proliferate, migrate, differentiate into myelinating oligodendrocytes, or remain quiescent. These fate decisions, in turn, remodel the myelin architecture of the CNS in ways that alter conduction velocity, circuit synchrony, and ultimately behavior.

Over the past decade, activity-dependent myelination has emerged as a central theme in research on neural plasticity, and the topic has been covered by several comprehensive reviews addressing both adaptive function in health and maladaptive changes in disease (Knowles et al., 2022). The present review is set apart from these earlier accounts by its synapse-centered, four-level framework, which traces the causal chain from neuron–OPC synaptic signaling through OPC fate decisions to myelin plasticity and behavioral output, explicitly separating established causal mechanisms from correlative or unresolved evidence at each level. This review organizes the OPC–synapse–myelin–behavior axis into a hierarchical framework spanning four levels of analysis (Figure 1), Within this axis, direct synaptic transmission offers the most spatially precise readout of local circuit activity, whereas extrasynaptic signaling by ambient neurotransmitter and activity-dependent paracrine factors broaden the range of neuronal signals that converge on OPC fate decisions; we maintain this distinction throughout the review. At the synaptic level, we describe the neurotransmitter receptor repertoire of OPCs and the forms of plasticity at neuron–OPC synapses, while critically evaluating the evidence for their functional significance. At the cellular level, we examine how synaptic signals are transduced into OPC fate decisions, emphasizing the distinction between *in vitro* and *in vivo* evidence and the challenges of attributing effects to specific receptor populations. At the circuit level, we discuss myelin plasticity as a structural output that modifies conduction velocity and network timing, critically appraising the human imaging evidence. Finally, at the behavioral level, we evaluate evidence that activity-driven myelination is required for specific forms of learning and memory, distinguishing studies that establish necessity from those that merely demonstrate correlation. We further consider how disruption of this axis contributes to neurological and psychiatric disease, with a frank assessment of the translational gap, and highlight key open questions for the coming decade.

**FIGURE 1:**
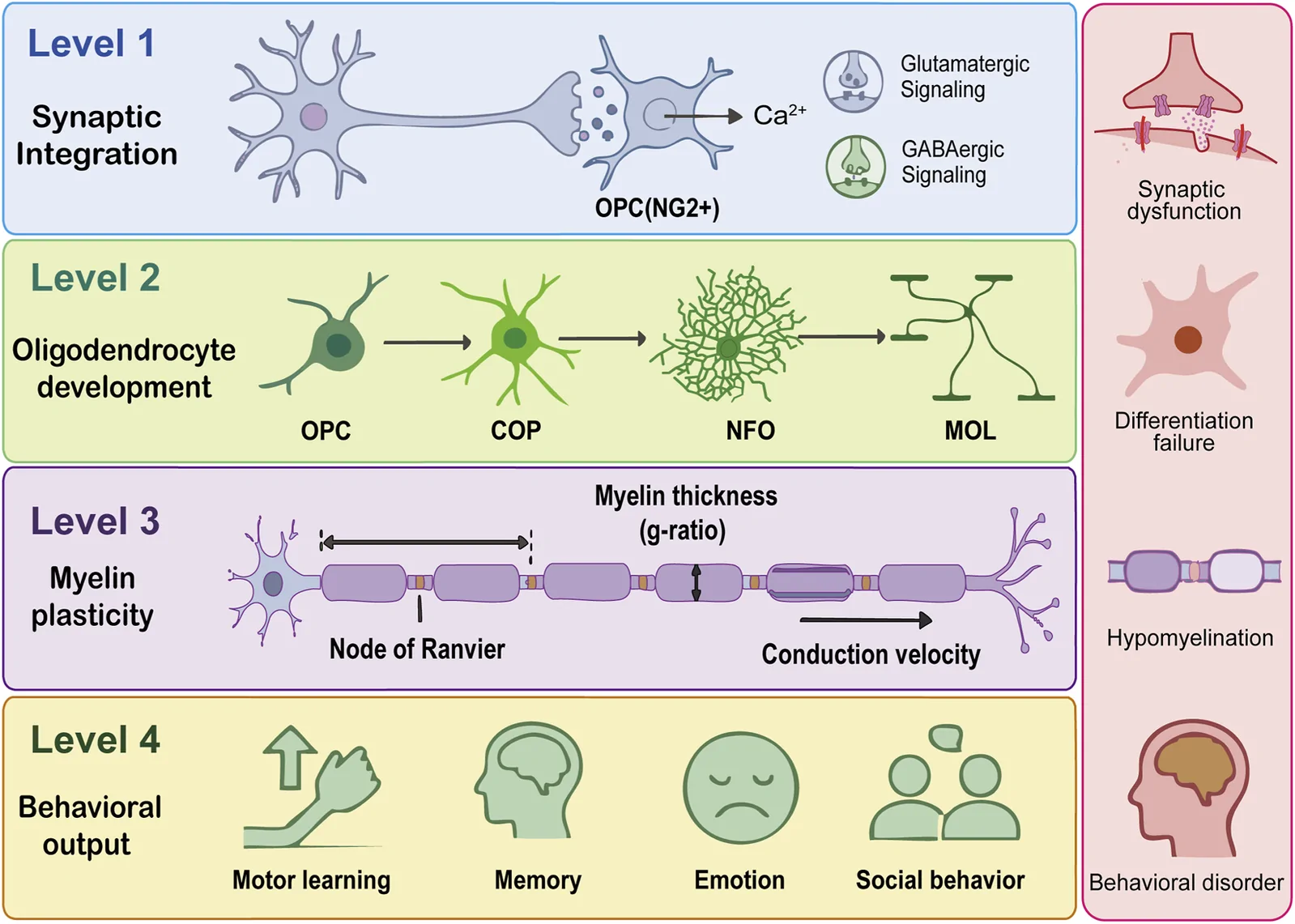
The OPC–synapse–myelin–behavior axis: a hierarchical framework. The figure organizes the biology reviewed in this article into four levels of analysis. Level 1 (synaptic): neuron–OPC synaptic contacts receive both glutamatergic inputs and GABAergic inputs. Level 2 (cellular): neurotransmitter receptor activation drives intracellular calcium dynamics and downstream transcriptional programs that govern OPC fate decisions. Level 3 (circuit): OPC differentiation produces myelin plasticity, defined as experience-dependent remodeling of internode length, sheath thickness (g-ratio), and nodal gap length, which adjusts axonal conduction velocity and circuit synchrony. Level 4 (behavioral): activity-driven myelination underlies specific behavioral capacities including motor skill acquisition, spatial memory consolidation, fear extinction learning, and social experience-dependent prefrontal circuit maturation. Abbreviations: OPC, oligodendrocyte precursor cell; COP, Committed Oligodendrocyte Precursor; NFO, Newly Formed Oligodendrocyte; MOL, Mature Oligodendrocyte.

## 2 The synaptic level: architecture and physiology of Neuron–OPC communication

### 2.1 Structural features of neuron–OPC synapses

Neuron–OPC synapses share several ultrastructural hallmarks with classical neuron–neuron synapses, including a presynaptic active zone with docked vesicles, a synaptic cleft, and a postsynaptic density apposed to the OPC membrane (Bergles et al., 2000). In the hippocampus, Schaffer collateral axons and local GABAergic interneurons form synapses onto OPC processes; In the cerebellum, OPCs also receive synaptic input from local axons, including parallel fibers (Lin and Bergles, 2004; Lin et al., 2005); and in the cortex, OPCs receive both glutamatergic and GABAergic synaptic inputs, with substantial contributions from GABAergic interneurons (Vélez-Fort et al., 2010). This anatomical specificity, in which OPCs sample inputs from the principal circuit elements of a given region, suggests that synapse formation is governed by circuit logic rather than random proximity.

OPC processes are co-inserted at some nodes of Ranvier (Serwanski et al., 2017; Li et al., 2024a), positioning these progenitors to sense both synaptic and periaxonal signals. Approximately 40% of cortical OPCs maintain close somatic proximity to GABAergic neurons, a configuration that allows synchronized postsynaptic currents and may serve a network surveillance function (Bai et al., 2021). Such architectural specificity implies that OPCs are not passive eavesdroppers but are integrated partners within defined microcircuits. However, electron microscopy cannot reveal the dynamics of OPC synapses; *in vivo* imaging shows that their contacts are largely transient (Hughes et al., 2013), so it remains unclear whether miniature excitatory postsynaptic currents (mEPSCs) recorded from OPCs originate from stable synapses or spillover signals, which is a distinction with important implications for intervention strategies.

### 2.2 Electrophysiological properties: a partially excitable progenitor

OPCs occupy a unique electrophysiological niche among glial cells. They exhibit a relatively hyperpolarized resting membrane potential (approximately −70 to −85 mV) and high input resistance, and receive both excitatory and inhibitory synaptic inputs, enabling them to integrate neuronal activity signals (Káradóttir et al., 2008). OPCs express voltage-gated Na^+^ channels that can generate action potential like sodium spikes, particularly during early postnatal development. While these spikes are unlikely to support long-range information transmission, they may amplify local depolarization in OPC processes at synaptic sites, enhancing calcium influx through voltage-gated calcium channels and calcium-permeable AMPARs (cpAMPARs) (De Biase et al., 2011).

As OPCs differentiate, voltage-gated Na^+^ and K^+^ channels are downregulated, input resistance falls sharply, and inwardly rectifying K^+^ channels (Kir) become prominent, a transition that suppresses excitability and promotes the electrical stability required for the energy-intensive process of myelin production (Hibino et al., 2010). This developmental electrophysiological trajectory reflects a fundamental shift in cellular role: from an activity-sensing progenitor to a metabolically committed myelinating cell.

### 2.3 Plasticity at Neuron–OPC synapses

A striking parallel between neuron–OPC and neuron–neuron synapses is the capacity for activity-dependent synaptic plasticity. High-frequency stimulation that drives calcium influx through cpAMPARs can induce long-term potentiation (LTP) at neuron–OPC synapses (Ge et al., 2006). Activation of metabotropic glutamate receptors (mGluRs) on OPCs modifies cpAMPAR surface expression, analogous to mGluR dependent LTD. at interneuron synapses (Zonouzi et al., 2011). GABAergic inputs to OPCs can transiently suppress AMPAR mediated currents, providing moment to moment modulation of excitatory drive (Lin and Bergles, 2004).

These bidirectional plasticity mechanisms imply that the neuron–OPC synapse is not a simple relay but a computational element capable of integrating patterns of circuit activity over multiple timescales. This property positions the synapse as a temporal filter that translates circuit-level dynamics into graded changes in OPC excitatory drive, which in turn modulate the probability and timing of progenitor differentiation (Figure 2). However, the functional impact of LTP- versus LTD-like mechanisms is likely shaped by the balance between glutamatergic and GABAergic signaling, which varies substantially across developmental stages and brain regions but remains incompletely characterized.

**FIGURE 2:**
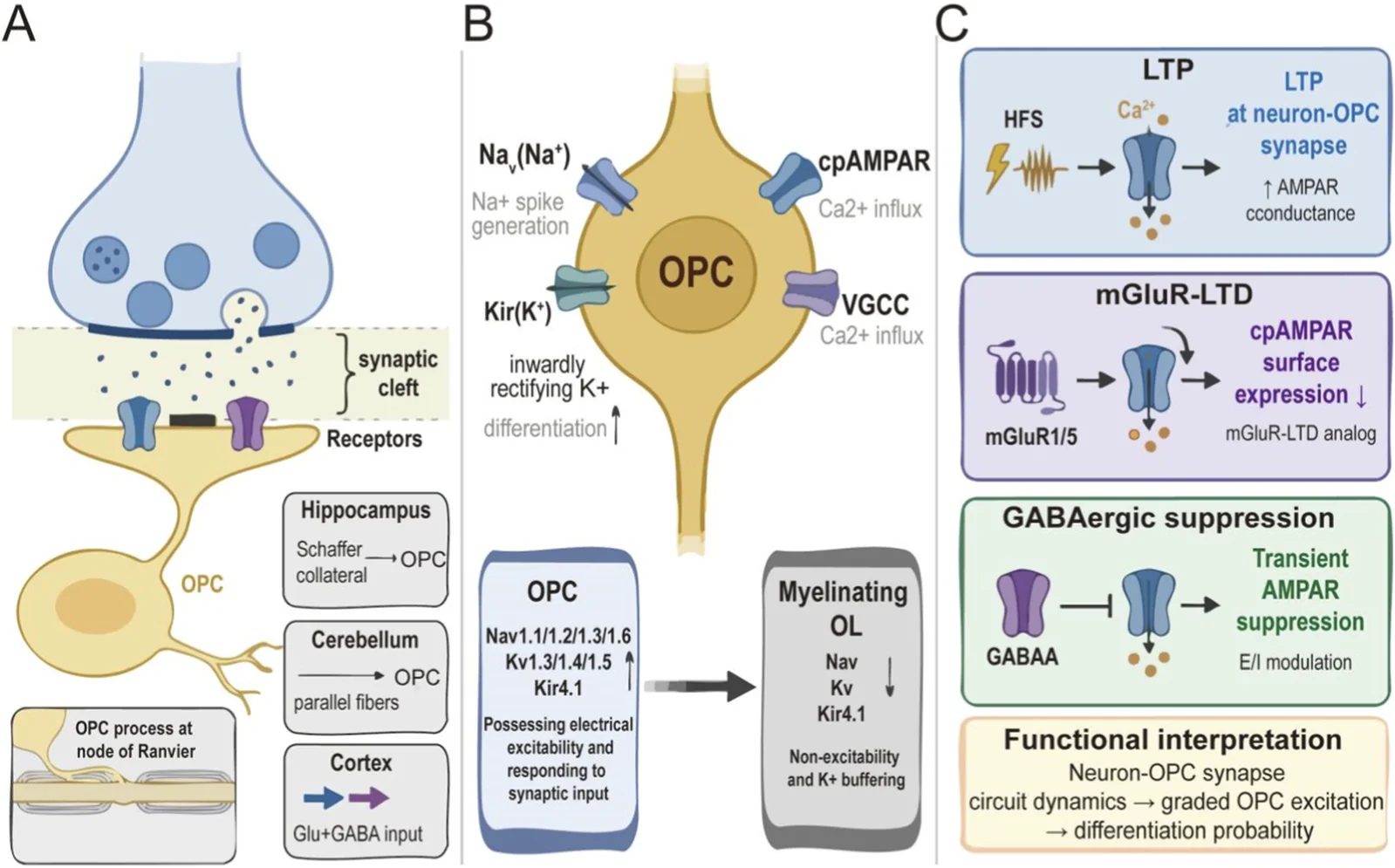
Synaptic architecture, electrophysiology, and plasticity at neuron–OPC synapses **(A)** Cross sectional schematic of a neuron-to-OPC synapse. OPC processes receive glutamatergic input from axons and GABAergic input from interneurons; these two classes of input are delivered by distinct presynaptic terminals and activate cpAMPARs and GABAA receptors, respectively, at the postsynaptic membrane of the OPC process **(B)** Central OPC Soma with labeled ion channel complement. Left membrane: voltage gated Na^+^ channels and inwardly rectifying K^+^ channels. Right membrane: cpAMPARs and voltage gated calcium channels (VGCCs) **(C)** Three stacked mechanism blocks. (I) High-frequency stimulation (HFS) drives cpAMPAR-mediated calcium influx, inducing LTP at neuron–OPC synapses. (ii) mGluR1/5 activation modifies cpAMPAR surface expression, representing a form of mGluR dependent LTD. analogous to mechanisms in hippocampal interneurons. (iii) GABAA receptor activation transiently suppresses AMPAR mediated currents, providing excitatory/inhibitory modulation. Abbreviations: OPC, oligodendrocyte precursor cell; cpAMPAR, calcium-permeable AMPA receptor; GABAA, gamma aminobutyric acid receptor type A; mGluR, metabotropic glutamate receptor; Nav, voltage gated sodium channel; Kir, inwardly rectifying potassium channel; VGCC, voltage gated calcium channel; HFS, high-frequency stimulation; LTP, long term potentiation; LTD., long term depression; E/I, excitatory/inhibitory.

## 3 Glutamatergic signaling: AMPA, NMDA, and metabotropic receptors

### 3.1 AMPA receptors: gateway to calcium dependent OPC regulation

AMPA receptors (AMPARs) are the primary mediators of fast glutamatergic signaling in OPCs. Critically, OPCs predominantly express calcium-permeable AMPARs (cpAMPARs), which are subunits lacking the RNA edited GluA2 (Ge et al., 2006). Calcium influx via cpAMPARs triggers downstream signaling cascades that inhibit OPC proliferation (Khawaja et al., 2021) and promote differentiation. Beyond ionotropic signaling, the GluA2 subunit acts as a molecular scaffold, interacting with alpha integrin and myelin proteolipid protein (PLP) to facilitate OPC migration (Gudz et al., 2006) (Figure 3).

**FIGURE 3:**
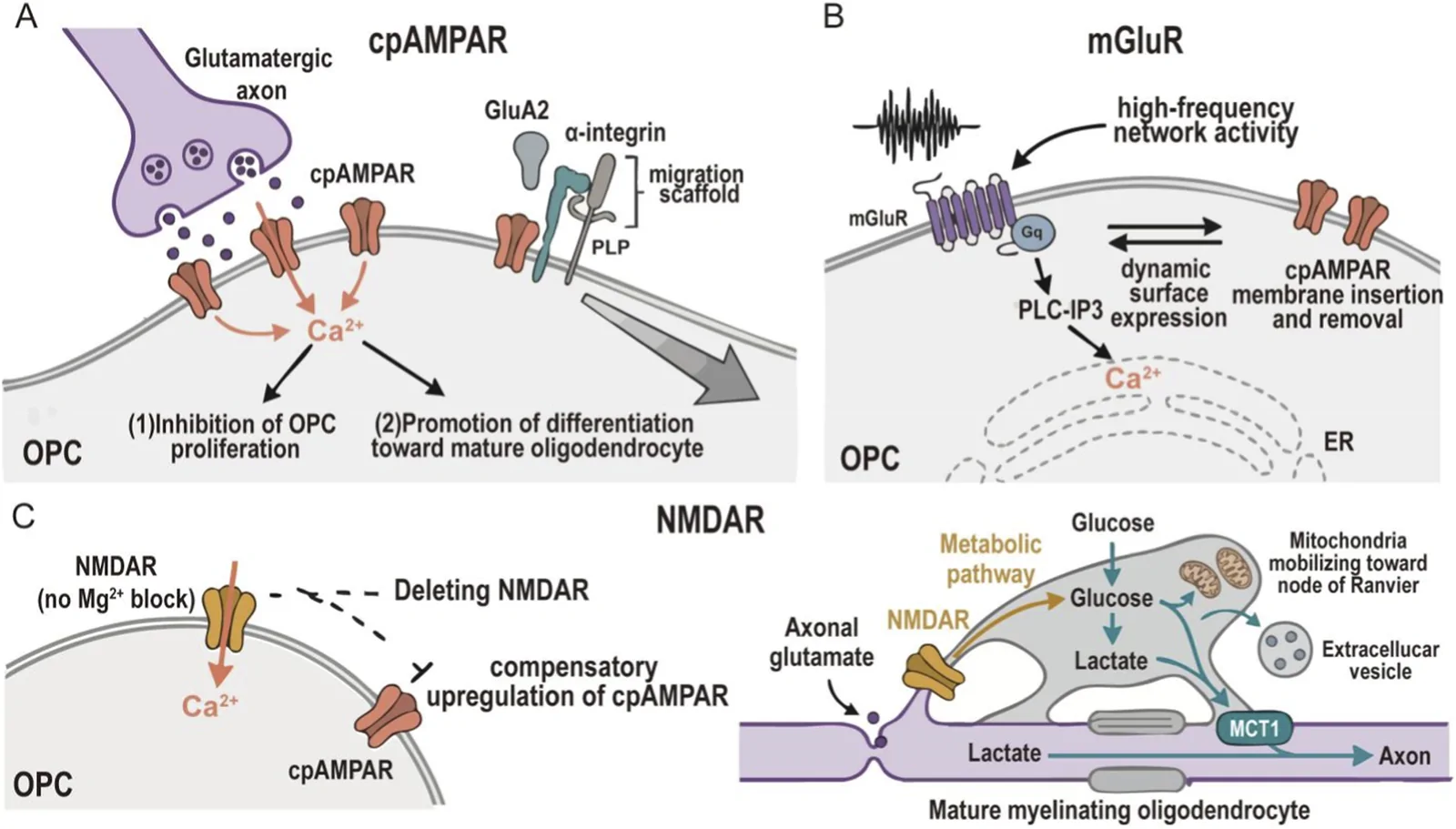
Glutamatergic signaling in OPCs **(A)** cpAMPARs in OPCs. OPCs receive glutamatergic input from axons via cpAMPARs (lacking the GluA2 subunit). Activation of these receptors leads to Ca^2+^ influx. Additionally, cpAMPARs interact with α-integrin and Proteolipid Protein (PLP) to form a migration scaffold that facilitates OPC motility **(B)** Metabotropic glutamate receptors (mGluRs) and homeostatic plasticity. High-frequency network activity triggers mGluRs. This activates the PLC-IP3 pathway, inducing Ca^2+^ release from the endoplasmic reticulum (ER) **(C)** NMDARs and metabolic support. In OPCs, NMDARs lack the typical Mg^2+^ block at resting membrane potentials, allowing Ca^2+^ entry. Genetic deletion of NMDARs leads to a compensatory upregulation of cpAMPARs, suggesting a homeostatic interplay between these receptor types. In mature myelinating oligodendrocytes, axonal glutamate activates NMDARs and triggers a metabolic pathway. This stimulates glucose uptake and its conversion to lactate, which is then transported to the axon via Monocarboxylate Transporter 1 (MCT1) to provide energetic support. Furthermore, NMDAR signaling promotes mitochondria mobilization toward nodes of Ranvier and the release of extracellular vesicles. Abbreviations: OPC, oligodendrocyte precursor cell; cpAMPAR, calcium-permeable AMPA receptor; mGluR, metabotropic glutamate receptor; NMDAR: N-methyl-D-aspartate receptor.

Recent *in vivo* genetic evidence has refined our understanding. Conditional deletion of GluA2/4, combined with GluA3 germline knockout, does not significantly impact OPC proliferation, but markedly reduces the density of mature oligodendrocytes, indicating that AMPAR signaling is specifically required for differentiation rather than progenitor maintenance (Kougioumtzidou et al., 2017). As OPCs transition toward a myelinating state, AMPAR mediated synaptic input is progressively downregulated, marking a developmental shift from electrically responsive progenitors to passive, myelinating cells (Zhang et al., 2014; Pivoňková et al., 2024). From a behavioral perspective, this trajectory implies a developmentally constrained window during which activity-dependent glutamate signaling can shape OPC fate, a consideration directly relevant to sensitive periods for learning and cognitive development.

Notably, these findings should be interpreted with an important caveat: cpAMPAR activation in OPCs can arise not only from direct neuron–OPC synaptic transmission, but also from glutamate spillover from nearby neuronal synapses. As a result, current genetic knockout studies cannot clearly distinguish synaptic from extrasynaptic cpAMPAR signaling. This distinction is important because if spillover alone is sufficient to promote OPC differentiation, neuron–OPC synapses themselves may not be essential for myelination. Resolving this issue will require approaches that selectively disrupt synaptic input onto OPCs while preserving ambient glutamate signaling. In addition, the existence of a developmental window for activity-dependent OPC regulation raises further questions about the timing and reversibility of these effects during brain maturation.

### 3.2 Metabotropic glutamate receptors: modulators of cpAMPAR expression

Group I metabotropic glutamate receptors (mGluR1/5) are expressed by OPCs, and their activation elevates intracellular calcium through release from IP3-sensitive stores (Luyt et al., 2003). Functionally, mGluR activation modifies cpAMPAR surface expression at neuron–OPC synapses (Zonouzi et al., 2011), representing a form of mGluR-dependent plasticity that parallels mechanisms observed in hippocampal interneurons. By dynamically adjusting the complement of calcium-permeable receptors at the postsynaptic membrane, mGluRs may serve as a gain control mechanism, amplifying or attenuating the calcium signals that determine OPC proliferative and differentiative responses to sustained glutamate release during high-frequency network activity.

A critical gap in this picture is that the downstream consequences of mGluR signaling for OPC development have been characterized primarily in dissociated cell cultures or acute slice preparations, where the concentration of agonists applied often exceeds physiological levels of extracellular glutamate. Whether mGluR-dependent modulation of cpAMPAR expression is engaged during natural patterns of circuit activity *in vivo*, and whether it produces effects on OPC differentiation that are independent of concomitant ionotropic AMPAR activation, have not been demonstrated. Given that mGluR1/5 antagonists have complex circuit-level effects, including on excitatory–inhibitory balance, pharmacological studies face inherent confounds. Conditional OPC-specific mGluR knockout models would provide more definitive evidence and represent an important direction for future work.

### 3.3 NMDA receptors: metabolic coupling and neuroprotection rather than differentiation

NMDARs are expressed by both OPCs and mature oligodendrocytes, although their subunit composition and functional properties differ from those of neurons. OPC NMDARs exhibit reduced Mg^2+^ block at physiological potentials, enabling activation without prior membrane depolarization (Káradóttir et al., 2005; Micu et al., 2006). Conditional deletion of the obligatory GluN1 subunit throughout the oligodendrocyte lineage leaves OPC survival, morphology, proliferation, differentiation, and myelination intact, indicating that NMDAR signaling is dispensable for the canonical progenitor life cycle (De Biase et al., 2011) (Figure 3) However, this dispensability may be context-dependent: neuregulin and BDNF have been reported to switch oligodendroglial myelination toward an NMDA receptor-dependent mode (Lundgaard et al., 2013), suggesting that NMDAR signaling can contribute to activity-dependent myelination when such cues are present.

The most compelling function of oligodendrocyte NMDARs is metabolic. Axonal glutamate release activates oligodendrocyte NMDARs, stimulating Ca^2+^ dependent glucose uptake and its conversion to lactate, which is then delivered to axons via monocarboxylate transporter 1 (MCT1) (Saab et al., 2016; Lee et al., 2012). NMDAR activation also mobilizes oligodendrocyte mitochondria toward nodes of Ranvier and triggers release of extracellular vesicles that provide further metabolic support to axons (Rinholm et al., 2016; Krämer-Albers and Werner, 2023). This metabolic coupling function has functional significance for axonal conduction and recovery under metabolic stress, as axons whose metabolic support is disrupted show impaired recovery from oxygen glucose deprivation, with implications for vulnerability to ischemic injury and neurodegeneration.

While the metabolic role of oligodendrocyte NMDARs is increasingly supported, most direct evidence linking NMDAR signaling to metabolic support has been obtained in mature myelinating oligodendrocytes rather than OPCs, leaving unresolved whether the same receptor populations regulate both axonal metabolic coupling and activity-dependent lineage progression. This distinction has significant translational implications, as therapies targeting NMDAR signaling could potentially alter axonal metabolic support without substantially affecting OPC differentiation or myelination. However, this possibility remains largely unexplored and will require validation in disease-relevant models.

## 4 GABAergic signaling: depolarizing inhibition and differentiation cues

### 4.1 Modes of GABA delivery to OPCs

GABA can influence OPCs through both direct synaptic input from interneurons and ambient extracellular GABA acting on extrasynaptic receptors. The relationship between interneurons and OPCs during development has been elucidated (Tian et al., 2025; Zhang et al., 2026; Yang et al., 2025; Li et al., 2026), and the synaptic GABAergic signaling to OPCs has also been well established (Lin and Bergles, 2004), with a relative shift toward extrasynaptic signaling as myelination progresses. Notably, OPCs and oligodendrocytes express the GABA synthesizing enzymes GAD65 and GAD67 and exhibit GABA immunoreactivity *in vitro*, suggesting the existence of autocrine or paracrine GABAergic signaling within the oligodendrocyte lineage (Bai et al., 2021; Zhang et al., 2021). The functional distinction between synaptic and extrasynaptic GABA signaling is important, but experimentally difficult to resolve. It remains unclear whether the OPC GABA-signaling-related phenotypes primarily arise from loss of synaptic input, impairment of extrasynaptic signaling, or a combination of both.

### 4.2 GABAA receptors: depolarizing calcium signals and behavioral consequences

In a developmental parallel to immature neurons, OPCs maintain elevated intracellular chloride concentrations through expression of NKCC1. Consequently, GABAA receptor activation leads to Cl^−^ efflux and membrane depolarization rather than hyperpolarization (Lin and Bergles, 2004). This depolarizing response has important downstream consequences, as GABA induced depolarization activates voltage gated Na^+^ channels, and the subsequent rise in intracellular Na^+^ reverses Na^+^/Ca^2+^ exchanger activity, elevating intracellular Ca^2+^ and promoting OPC migration (Kirchhoff and Kettenmann, 1992). GABA evoked depolarization also activates VGCCs directly, causing Ca^2+^ influx and promoting release of BDNF, which in turn supports OPC proliferation (Kirchhoff and Kettenmann, 1992).

The behavioral importance of this pathway is illustrated by the γ2 subunit knockout. Conditional deletion of the GABAA receptor γ2 subunit in OPCs during early postnatal development disrupts interneuron-OPC synaptic transmission and causes hypomyelination of parvalbumin-positive (PV+) interneurons in the barrel cortex (Benamer et al., 2020; Plaisier et al., 2025). Because PV + interneurons drive gamma oscillations essential for sensory processing and cognitive function, this hypomyelination has direct implications for the circuit maturation that underlies sensory cognitive development, a finding of direct relevance to neurodevelopmental disorders characterized by PV interneuron hypofunction, such as schizophrenia.

### 4.3 GABAB receptors: metabotropic modulation of differentiation

GABAB ​ receptors are expressed across the oligodendrocyte lineage. Their activation is coupled to Gαi/o proteins, leading to inhibition of adenylyl cyclase, reduced cAMP, and suppression of PKA activity (Luyt et al., 2007). Reduced PKA signaling attenuates CREB-dependent transcriptional programs, modulating expression of genes involved in neural development including BDNF and glutamate receptor subunits (Figure 4). In primary cultures, GABAB receptor activation promotes OPC differentiation and enhances myelin protein expression via Src-family kinase signaling (Serrano-Regal et al., 2020). The GABAB-cAMP/PKA/CREB axis thus provides a metabotropic complement to the ionotropic GABAA pathway: whereas GABAA receptors drive acute calcium signals regulating migration and local BDNF release, GABAB receptors modulate the transcriptional programs that determine differentiation competence over longer timescales. More broadly, glutamatergic and GABAergic signaling regulate OPC fate through distinct mechanisms and on different timescales. Glutamatergic signaling, driven largely by axonal glutamate release, delivers activity-dependent calcium signals through cpAMPARs that inhibit proliferation and promote OPC differentiation, whereas GABAergic signaling, from both interneuron synapses and ambient tone, acts through depolarizing GABAA and metabotropic GABAB receptors to regulate OPC migration, proliferation, and differentiation. These pathways also interact on a fast timescale, as GABAergic input can transiently suppress AMPA receptor-mediated currents in OPCs. Over longer timescales, GABAergic signaling exerts its effects through calcium/BDNF signaling downstream of GABAA receptors and cAMP/PKA/CREB signaling downstream of GABAB receptors, indicating that the balance of glutamatergic and GABAergic input shapes how OPCs integrate circuit activity into fate decisions.

**FIGURE 4:**
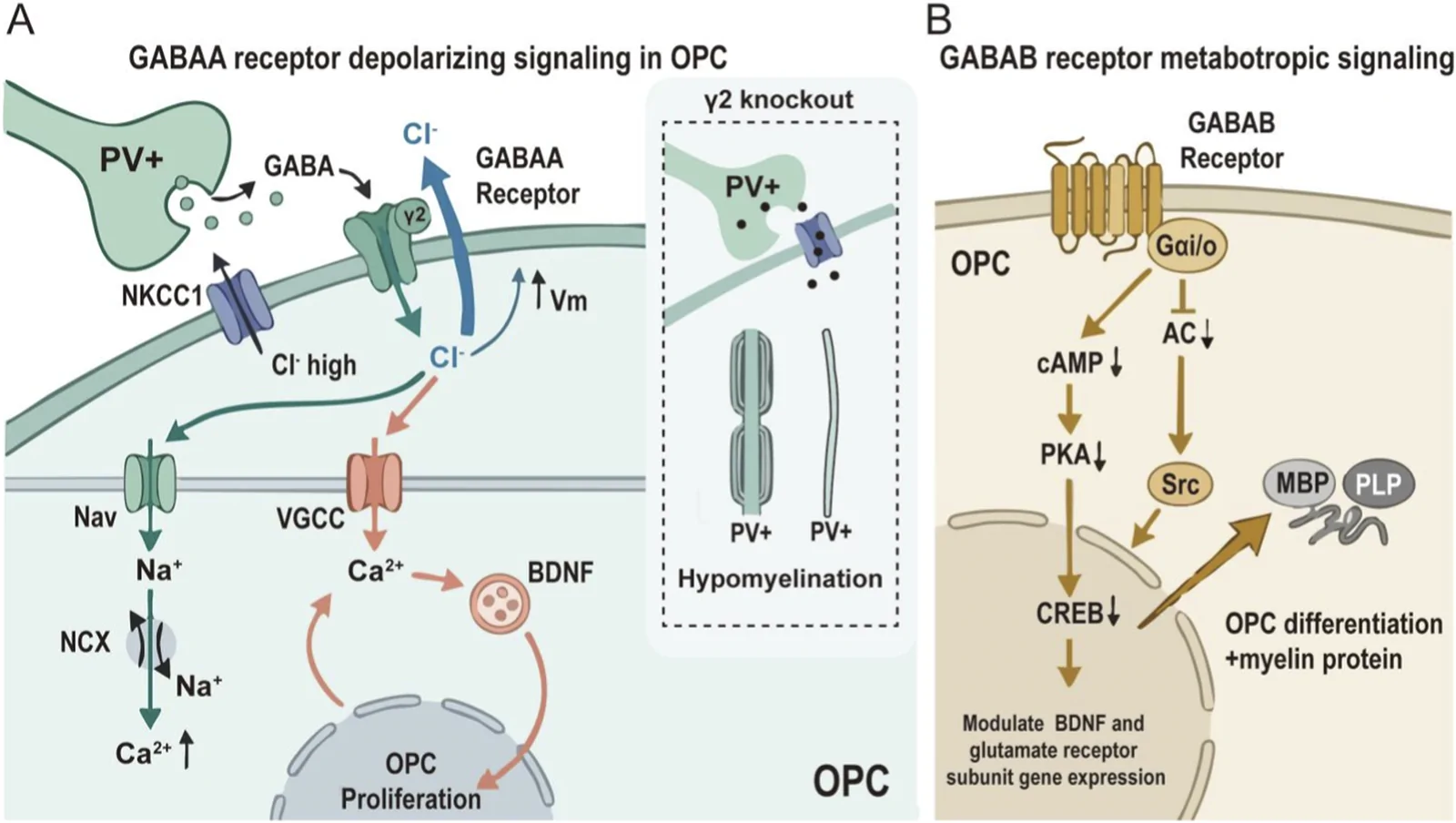
GABAergic signaling in OPCs **(A)** GABAA receptor-mediated depolarizing signaling. OPCs receive GABAergic input from PV + interneurons. High intracellular Cl^−^ concentrations, maintained by the transporter NKCC1, result in Cl^−^ efflux upon GABAA receptor activation, leading to membrane depolarization. This depolarization activates voltage gated channels. The resulting Ca^2+^ influx, further amplified by the sodium calcium exchanger (NCX), triggers the release of BDNF and promotes OPC proliferation **(B)** GABAB receptor-mediated metabotropic signaling. Activation of G-protein coupled GABAB receptors inhibits AC, leading to a decrease in cAMP levels and PKA activity. This cascade suppresses CREB phosphorylation, which modulates the gene expression of BDNF and glutamate receptor subunits. Concurrently, GABAB receptor signaling through Src kinases promotes OPC differentiation and the synthesis of myelin proteins, such as MBP and PLP. Abbreviations: AC: Adenylate cyclase; BDNF: Brain-derived neurotrophic factor; NCX: Sodium-calcium exchanger; PKA: Protein kinase A; VGCC: Voltage-gated calcium channel.

## 5 The cellular level: activity-dependent OPC fate decisions

### 5.1 Neuronal activity as a regulator of proliferation and differentiation

Seminal experiments by Barres and Raff first established that electrical activity in axons promotes myelination, a finding now supported by an extensive body of evidence (Barres and Raff, 1993) (Figure 5). Optogenetic stimulation of motor cortex neurons elicits a dramatic, region-specific increase in OPC proliferation, differentiation, and myelination of subcortical axons belonging to the stimulated population, accompanied by altered gait patterns, demonstrating that activity driven myelination produces functionally meaningful modifications of circuit output (Gibson et al., 2014). This paradigmatic finding established two key principles: first, that neural activity drives oligodendrogenesis; and second, that the behavioral consequences of this plasticity are measurable.

**FIGURE 5:**
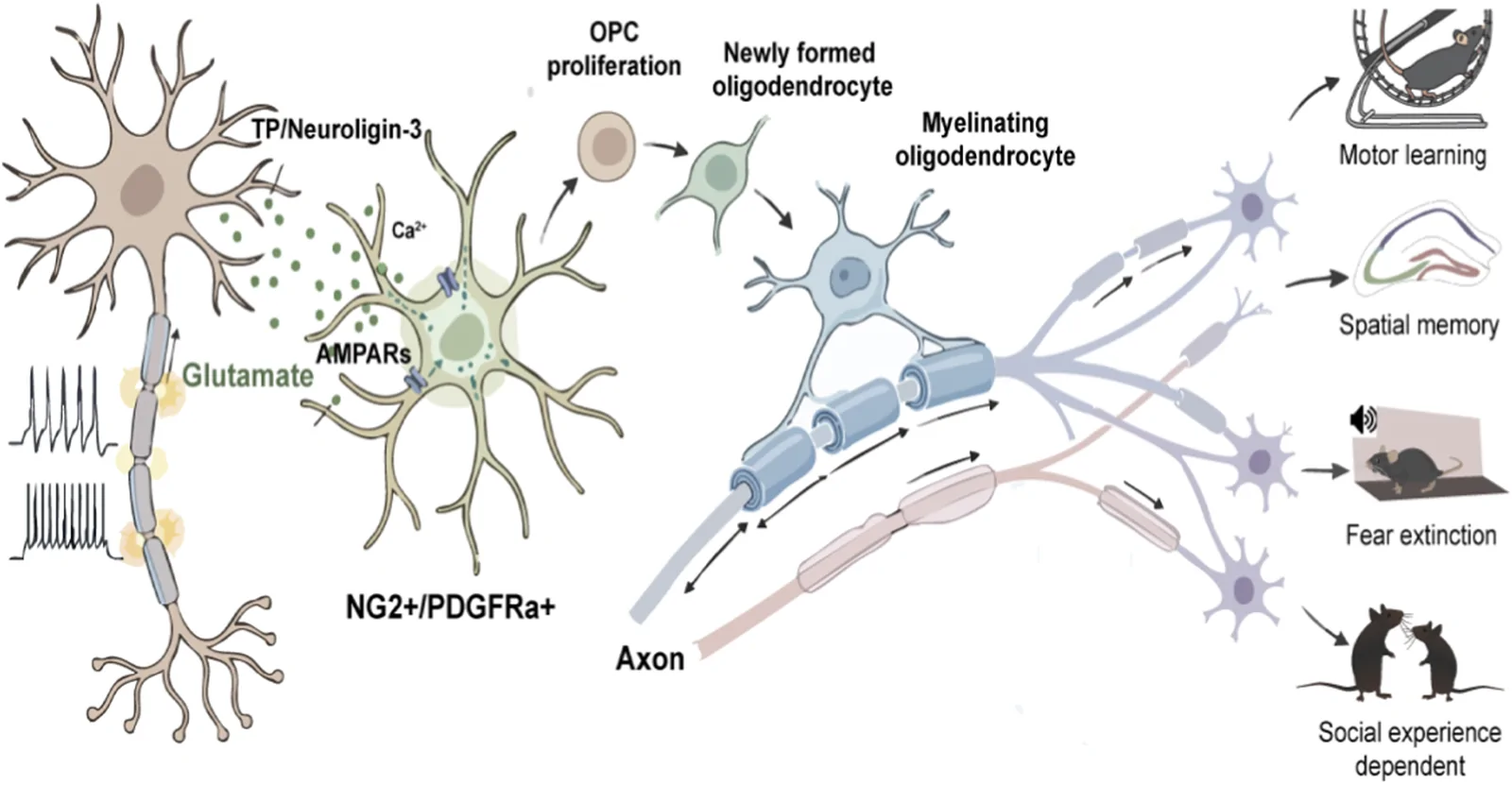
Neuronal activity–OPC–myelin coupling links cellular plasticity to circuit function and behavior. Schematic illustrating how neuronal activity regulates OPC fate and activity-dependent myelin remodeling. Changes in myelin structure fine-tune axonal conduction and circuit synchrony, thereby supporting behavioral plasticity, including motor learning, spatial memory, fear extinction, and social adaptation.

Neuronal activity regulates OPC behavior through multiple, partially redundant mechanisms. Active neurons release glutamate, ATP derived adenosine, and neuroligin-3, all of which influence OPC development (Gautier et al., 2015). Activity-dependent cleavage and secretion of neuroligin-3 stimulates OPC proliferation via PI3K–mTOR signaling. Changes in extracellular K^+^ associated with neuronal firing modulate OPC membrane properties through voltage gated potassium channels, influencing their functional state (Maldonado et al., 2013). Astrocytes play a key intermediary role by responding to neuronal activity with ATP release, which activates purinergic signaling in OPCs, and by providing metabolic support through glial networks (Niu et al., 2016). This multimodal signal integration positions OPCs as sensitive activity detectors capable of responding to graded changes in circuit state across a wide dynamic range.

A critical limitation of the optogenetic studies that most convincingly demonstrate activity-dependent myelination is that optogenetic stimulation often drives neuronal firing patterns that may not fully recapitulate physiological circuit activity. As a result, multiple activity-dependent signals, including glutamate, adenosine, extracellular K^+^, and potentially other released factors, are simultaneously engaged, making their individual contributions difficult to disentangle. For example, Gibson et al. (Gibson et al., 2014) showed that optogenetic stimulation of premotor cortex neurons promotes OPC proliferation and differentiation, yet the specific receptor pathways mediating these effects were not identified. Consequently, although neuronal activity clearly influences myelination, it remains unresolved which signaling pathways are mechanistically required, as opposed to simply co-activated during increased circuit activity. Resolving this issue will require approaches that combine cell-type-specific receptor manipulation with controlled activity paradigms and *in vivo* monitoring of OPC response.

### 5.2 Vesicular release and calcium transients: axon level selection for myelination

Elegant zebrafish studies have established that vesicular neurotransmitter release from individual axons influences which axons are selected for myelination. Blocking vesicle fusion using tetanus toxin causes oligodendrocytes to more frequently retract nascent sheaths from those axons and preferentially myelinate neighboring active axons (Hines et al., 2015). These stabilization and retraction decisions are associated with local calcium transients in OPC processes, where processes that ultimately form sheaths show sustained calcium signals, while those that retract exhibit transient or absent calcium activity (Li et al., 2024a; Baraban et al., 2018; Krasnow et al., 2018). This calcium guided model implies that synaptically released glutamate activates cpAMPARs locally in OPC processes, and that these calcium signals act as binary decision signals to initiate or abort the myelination program at specific axonal contacts.

Importantly, the requirement for vesicular release is axon type specific. In zebrafish, tetanus toxin expression in reticulospinal neurons reduces myelination, while the same manipulation in commissural primary ascending neurons does not alter myelination (Call et al., 2026; Koudelka et al., 2016). This axon type specificity implies that the rules governing activity-dependent myelination are not universal but are tuned to the identity and functional demands of specific axon populations, a principle with important implications for understanding region-specific white matter development and vulnerability. Notably, individual oligodendrocytes in zebrafish myelinate fewer axons than their mammalian counterparts, which may make axon-selection decisions more experimentally tractable; however, further validation in mammals is required.

### 5.3 Bidirectional OPC–Neuron communication

The adult CNS maintains a large population of OPCs that do not differentiate, yet preserve stable densities, express neurotransmitter receptors, and maintain synaptic contacts with neurons. The persistent synaptic connectivity of adult OPCs raises the possibility that these cells serve constitutive functions in circuit homeostasis beyond myelin replacement (Yi et al., 2023). OPCs release the neuromodulatory pentraxin Nptx2 (Narp), which modulates neuronal AMPARs and thus circuit excitability (Moura et al., 2021). OPCs also release growth factors including NGF and BDNF that support neuronal health (Wilkins et al., 2003), and participate in synaptic pruning and structural remodeling of neural circuits (Auguste et al., 2022; Buchanan et al., 2022).

The characterization of adult OPCs as constitutively active participants in circuit homeostasis, rather than merely quiescent reserve progenitors, represents a significant conceptual revision. However, this picture is based on a relatively small number of studies that have often used *in vitro* or acute preparation approaches. Whether the signaling molecules identified (Nptx2, NGF, BDNF) are released at physiologically relevant concentrations *in vivo*, and whether their synaptic effects are quantitatively significant compared to the much larger pools secreted by neurons themselves, has not been rigorously established. The engulfment of synapses and axonal material by OPCs is perhaps the most striking non-myelination function yet reported, but the signals that regulate the selectivity of this engulfment remain poorly understood. Systematic *in vivo* genetic perturbation of OPC secretory and phagocytic functions, combined with comprehensive circuit-level phenotyping, will be essential for establishing the importance of these processes.

## 6 Circuit and behavioral levels: myelin plasticity as adaptive output

### 6.1 Structural dimensions of myelin plasticity

Myelin plasticity encompasses several independently regulated structural parameters, including the number of myelin internodes per oligodendrocyte, internode length, myelin sheath thickness (g-ratio), and nodal length (Figure 5). Each parameter has well-defined consequences for axonal conduction velocity and the metabolic cost of impulse propagation (Arancibia-Cárcamo et al., 2017). A single oligodendrocyte can myelinate multiple axons, and the properties of individual sheaths can be regulated independently, allowing fine grained spatial control over conduction in heterogeneous axon populations. These structural degrees of freedom provide the substrate for a rich and flexible plasticity capable of adapting circuit timing to experience.

Human neuroimaging provided the first evidence that myelin is dynamic in adults. MRI diffusion tensor imaging (DTI) and magnetization transfer studies document white matter changes in response to skill acquisition, with increases in fractional anisotropy (FA) and myelin volume fraction reported after piano practice (Bengtsson et al., 2005), juggling (Scholz et al., 2009), and other motor and cognitive training paradigms. These macroscale observations established the behavioral relevance of myelin plasticity and motivated the preclinical dissection of its cellular and molecular mechanisms.

A persistent and underappreciated challenge for this field is that DTI-derived metrics such as FA are not specific measures of myelin content. FA reflects the directionality of water diffusion within a voxel and can be affected by changes in axon diameter, axon density, crossing fibers, inflammation, and edema—all of which can change with learning-related activity independent of any change in myelin (Jones et al., 2013). Magnetization transfer ratio (MTR) is more specific for myelin content but is still confounded by changes in axon packing and water content. Quantitative MRI approaches such as myelin water fraction (MWF) and macromolecular proton fraction (MPF) provide greater specificity but have limited resolution and signal-to-noise in deep white matter structures. Consequently, the interpretation of human DTI studies as evidence for activity-driven myelination must be regarded as suggestive rather than definitive, and the strong mechanistic conclusions drawn from them should be tempered by these methodological limitations. The disconnect between the spatial resolution of human neuroimaging and the cellular scale at which myelin remodeling occurs remains a fundamental translational challenge.

### 6.2 Motor and spatial learning: oligodendrogenesis as a behavioral requirement

When mice are trained on a complex running wheel with irregularly spaced rungs, accelerated oligodendrocyte production is observed, and conditional deletion of the oligodendrocyte specific transcription factor *Myrf*, which blocks differentiation, impairs acquisition of the complex motor skill while leaving simple locomotion intact (McKenzie et al., 2014; Shimizu et al., 2023). A subpopulation of *Enpp6* ^+^ premyelinating oligodendrocytes is generated within hours of motor task exposure (Xiao et al., 2016), and this rapid progenitor response is *Myrf* dependent and necessary for skill acquisition, establishing that an early wave of oligodendrogenesis is a required step in the cellular cascade linking neural activity to behavioral adaptation. It should be noted, however, that *Myrf* acts downstream of OPC differentiation, and its deletion abolishes all aspects of oligodendrogenesis; these studies therefore demonstrate that an early wave of oligodendrogenesis is required for motor learning, but they do not directly test whether neuron–OPC synaptic signaling is the underlying causal mechanism.

Motor skill learning elicits retraction of pre-existing myelin sheaths and increased nodal length within 2 weeks, followed by formation of new sheaths on newly activated axons (Wang S. et al., 2025; Xin and Chan, 2022). New myelin formed after motor learning depends specifically on sphingomyelin and galactosylceramide synthesis, and ablation of these lipid pathways disrupts skill acquisition (Kato et al., 2023). For spatial memory, learning induced production of new oligodendrocytes in hippocampal cortical circuits myelinates newly potentiated circuit elements, increasing synchrony between hippocampus and cortex during memory consolidation; genetic impairment of oligodendrogenesis disrupts not only memory performance but also the cortico-hippocampal coupling that is its functional signature. Additionally, the number of new oligodendrocytes added during motor learning is a small fraction of the total oligodendrocyte population, and the specific axonal pathways that become newly myelinated have not been comprehensively mapped; the circuit mechanism by which a small increment in new myelin produces a measurable change in task performance therefore remains to be established.

### 6.3 Emotional learning, fear, and experience-dependent maturation

Fear conditioning and other forms of associative memory require OPC proliferation and differentiation, particularly in the prefrontal cortex; genetic blockade of oligodendrogenesis impairs fear memory extinction (Plaisier et al., 2025; Pan et al., 2020; Steadman et al., 2020).

Social deprivation during the critical period for oligodendrogenesis reduces myelination in the prefrontal cortex, accompanied by decreased MBP and MAG expression, shorter oligodendrocyte processes, and fewer internodes (Makinodan et al., 2012). This experience-dependent hypomyelination may involve an activity regulated vascular endothelin–EDNRB–PKCε signaling pathway, in which neuronal activity modulates vascular endothelin release to control oligodendrocyte myelinating capacity (Swire et al., 2019). Together, these findings establish that OPC driven myelin plasticity is a neural substrate for the enduring effects of early social experience on prefrontal circuit maturation and behavior, a finding directly relevant to developmental psychiatry.

### 6.4 Glial contributions to myelin remodeling

Myelin plasticity is not exclusively oligodendrocyte-driven; astrocytes and microglia make essential contributions. Perinodal astrocytes regulate paranodal integrity and nodal gap length through VAMP2 dependent exocytosis of the thrombin inhibitor protease nexin-1 (PN1), inhibiting thrombin mediated cleavage of the paranodal adhesion complex and maintaining optimal conduction velocity (Dutta et al., 2018). Microglia preserve myelin health through phagocytosis of myelin debris during normal remodeling, and during early development sculpt the final pattern of myelinated axons through recognition of phosphatidylserine on the myelin membrane (McNamara et al., 2023).

The recognition that astrocytes and microglia are active regulators of myelin structure represents an important conceptual advance, but it also introduces complexity that is often understated in the OPC-centric literature. The behavioral plasticity supported by myelination depends on the concerted action of the entire glial community, and interventions targeting OPC differentiation will inevitably perturb astrocyte and microglial function as well. For example, inflammatory microglial activation—which accompanies many learning paradigms, stress exposures, and disease states—simultaneously promotes myelin debris clearance and releases cytokines that inhibit OPC differentiation (Qiu et al., 2026). Disentangling the direct effects of OPC fate changes from the indirect effects mediated through changes in astrocyte and microglial state is a major methodological challenge that is inadequately addressed in current literature (Wang Z. et al., 2025). Future studies should incorporate comprehensive multiglial profiling alongside behavioral and electrophysiological phenotyping.

## 7 Pathological implications: disruption of the OPC–synapse–myelin–behavior axis

### 7.1 Multiple sclerosis: convergent failure of synaptic signaling and remyelination

Multiple sclerosis (MS) is characterized by focal immune mediated demyelination and failure of remyelination, partly due to impaired recruitment and differentiation of OPCs. The OPC synapse is disrupted at multiple levels in MS. Within active lesions, reactive astrocytes, infiltrating macrophages, and inflammatory cytokines alter the perisynaptic microenvironment, disrupting the glutamatergic and GABAergic inputs that normally sustain OPC differentiation competence. Glutamate excitotoxicity, mediated in part through OPC cpAMPARs, may paradoxically impair the very cells required for remyelination. At the network level, GABAergic signaling is profoundly disrupted in MS (Macnair et al., 2025). Electrophysiological studies in experimental autoimmune encephalomyelitis (EAE) demonstrate reduced spontaneous GABAergic currents and loss of inhibitory interneurons (Rossi et al., 2011), while *in vivo* MRS reveals decreased GABA levels in multiple brain regions.

The behavioral consequences of this combined synaptic and myelin pathology are substantial and reflect the circuit level disruptions. Cognitive impairment, mood dysregulation, and fatigue in MS correlate with white matter abnormalities in prefrontal and limbic circuits, consistent with disruption of the activity-dependent myelination that supports these circuits. These findings carry direct therapeutic implications. Pharmacological strategies that restore OPC synaptic input, for example, through GABAB agonism, or that protect OPC cpAMPARs from excitotoxic overactivation may represent viable approaches to enhancing remyelination in active MS lesions.

### 7.2 Psychiatric disorders: myelin plasticity as a behavioral substrate

Schizophrenia, bipolar disorder, and major depression are consistently associated with white matter abnormalities and reduced oligodendrocyte lineage cell density in prefrontal and limbic circuits. Traditionally, these alterations have been interpreted as secondary consequences of neuronal dysfunction. However, accumulating evidence now challenges this view by positioning oligodendrocyte precursor cells (OPCs) as active regulators of synaptic and circuit integrity.

In a schizophrenia disease model, pathological OPCs directly suppress synaptogenesis (Birey et al., 2015), while recent human genetic and cellular studies further demonstrate that disease associated OPC states are sufficient to induce synaptic deficits and schizophrenia like behavioral abnormalities (Yu et al., 2022). Crucially, both studies provide functional gain and loss of function evidence rather than merely correlative observations, supporting a revised framework in which aberrant OPC–neuron communication constitutes a primary driver of circuit dysfunction rather than a downstream consequence.

At the molecular level, chromatin remodeling mechanisms provide an additional layer of regulation linking oligodendroglial development to circuit integrity. The chromatin remodeler BAF155 coordinates transcriptional programs involved in both synaptic interaction and myelination. Its disruption leads to region-specific myelination deficits, synaptic abnormalities, and autism like behavioral phenotypes in mice (Wang X. et al., 2025), highlighting a convergence between epigenetic regulation, oligodendrocyte function, and neurodevelopmental circuit pathology.

The behavioral relevance of activity-dependent myelination is most directly demonstrated in fear related paradigms, making PTSD a particularly compelling application of the OPC–synapse–myelin–behavior framework. OPC differentiation in the prefrontal cortex is required for the preservation of remote fear memories: mice unable to form new myelin exhibit deficient remote, but not recent, fear memory recall, whereas pharmacological induction of myelination with clemastine improves remote recall and promotes fear generalization (Pan et al., 2020). The increased fear generalization associated with enhanced myelination may be of particular relevance to the proposed connection with PTSD, given that generalized fear is a core feature of the disorder. These findings carry direct translational significance. If OPC differentiation is a necessary step in the neural circuit changes that support fear extinction, then factors that impair oligodendrogenesis, including stress, neuroinflammation, or genetic vulnerability, may disrupt the extinction learning that is the cellular target of exposure-based psychotherapies.

It is important, however, to note two limitations of the current evidence base. First, the key studies in this domain use conditional oligodendrocyte specific knockouts, for example, *Myrf* deletion, which simultaneously abolish all aspects of OPC differentiation and therefore cannot isolate the contribution of synaptic signaling *per se* from the broader consequences of blocked myelination. Second, direct evidence in human PTSD, whether from *post mortem* cellular analyses, genetic association studies, or neuroimaging of myelin-specific markers, remains sparse. The mechanistic case is thus more compelling in rodent models than in clinical populations, and the translational gap warrants explicit acknowledgment.

## 8 Conclusion

The past 2 decades have established OPCs as sophisticated cellular integrators of neural circuit state, capable of detecting neurotransmitter signals at *bona fide* synapses and translating these signals into cell fate decisions that remodel the myelin architecture of the CNS. We have organized this biology into a hierarchical framework spanning four levels: the synaptic architecture and plasticity of neuron–OPC connections; the cellular mechanisms by which synaptic signals govern OPC fate; the structural dimensions of myelin plasticity as a circuit-level output; and the behavioral consequences of activity-driven myelination for motor learning, spatial memory, fear conditioning, and social experience-dependent development.

However, the field has progressed far more rapidly at the level of phenomenological description than at the level of mechanistic dissection of causal necessity. Is OPC synaptic signaling necessary for activity-driven myelination, or merely sufficient? Which molecular pathway is rate-limiting for sheath stabilization—cpAMPAR-mediated calcium, NMDAR activation, or extrasynaptic/paracrine signals? Is new myelin *per se* the behavioral effector in learning and memory paradigms, or do the metabolic and neurotrophic functions of newly differentiated oligodendrocytes account for some or all of the behavioral phenotype? These questions cannot be resolved by the experimental approaches that currently dominate the field.

Beyond these mechanistic gaps, several broader questions remain unresolved. The functional significance of neuron–OPC synapses in the adult brain is still unclear, particularly given that most adult OPCs do not actively differentiate, which raises the possibility that synaptic inputs regulate not only latent differentiation potential but also constitutive roles in circuit homeostasis, neuroprotection, and modulation of neuronal excitability. Increasing evidence for transcriptional heterogeneity among OPCs introduces an additional layer of complexity, as it remains unknown whether distinct OPC subtypes differ in their synaptic connectivity, receptor composition, or responsiveness to neurotransmitter signals. Recent studies further suggest that the functional diversity of oligodendrocyte-lineage cells may be rooted in their developmental history and transcriptional state. *Olig1* and *Olig2* have been shown to coordinately regulate cortical progenitor fate, promoting OPC specification while suppressing alternative interneuron progenitor programs, thereby establishing distinct developmental trajectories for cortical oligodendrocyte production (Tian et al., 2025; Li et al., 2024b). Consistent with a broader role for these transcriptional programs in glial maturation, *Olig1/2* also regulate cortical astrocyte maturation through direct modulation of BMP7 signaling, indicating that Olig-dependent transcriptional networks can shape the maturation state and functional properties of distinct glial populations (Wang Z. et al., 2025). At the level of cortical radial glia, integrated ERK/PKA, YAP/TAZ, and SHH signaling further defines lineage identity and biases progenitors toward neuronal, astrocytic, or oligodendroglial fates, with evidence for conservation of these regulatory principles across mouse and human cortex (Zhang et al., 2026; Yang et al., 2025; Gao et al., 2025). Importantly, analysis of the gyrencephalic ferret cortex extends this framework to a species with expanded cortical architecture and reveals conserved as well as regionally specialized radial glial programs associated with neurogenesis and gliogenesis (Li et al., 2026). Together, these findings raise the possibility that differences in OPC synaptic connectivity, receptor composition, and activity responsiveness may reflect, at least in part, distinct developmental origins and transcriptional states rather than representing a uniform property of the OPC population.

At the circuit level, the mechanisms that couple local calcium signals in OPC processes to the initiation and termination of individual myelin sheaths remain poorly defined, limiting our understanding of how activity-dependent signals are translated into precise patterns of myelin remodeling. Ultimately, these unresolved questions converge at the behavioral level, where activity-dependent myelination has been well characterized in motor and spatial learning but remains largely unexplored in higher cognitive domains such as language, social cognition, and emotional regulation. Clarifying these links, including the extent to which myelin plasticity contributes to the efficacy of interventions such as physical exercise, cognitive training, and psychotherapy, which will be essential for establishing a unified mechanistic framework.

Looking forward, the most productive directions for the field are likely to involve (Bergles et al., 2000): *in vivo* calcium imaging of OPC processes during defined learning paradigms in adult animals, to directly test whether the calcium-guided sheath stabilization model demonstrated in larval zebrafish operates in the adult mammalian brain (Knowles et al., 2022); development of OPC-specific conditional knockouts for individual receptor components to establish which pathways are necessary versus redundant (Lin and Bergles, 2004); transcriptomic and functional characterization of OPC heterogeneity, to determine whether distinct OPC subtypes differ in their synaptic connectivity and responsiveness to learning-related activity (Lin et al., 2005); development of myelin-specific MRI biomarkers with sufficient resolution and specificity to track adaptive myelination in the human brain; and (Vélez-Fort et al., 2010) direct functional testing of OPC-targeted therapeutic strategies in disease-relevant human cell systems and animal models, with explicit power analyses and pre-registration to guard against false positives in this rapidly moving field.

Ultimately, the OPC–synapse–myelin–behavior axis represents a previously underappreciated dimension of neural plasticity that complements and interacts with synaptic plasticity at neuron–neuron connections. Disruption at any level of this axis produces measurable behavioral consequences and contributes to the pathophysiology of disorders ranging from multiple sclerosis to psychiatric disease. As imaging, genetic, and electrophysiological tools continue to advance, the coming decade is likely to resolve the key causal questions identified in this review, define the full scope of myelin plasticity across the lifespan, and ultimately establish whether therapeutic targeting of the OPC–synapse–myelin axis can be translated from experimental models to human disease.
